# Tetra­aqua­bis­[4-(4*H*-1,2,4-triazol-4-yl)benzoato-κ*N* ^1^]manganese(II) deca­hydrate

**DOI:** 10.1107/S1600536811025335

**Published:** 2011-07-09

**Authors:** Ying-Ai Piao, Zhen-Yu Xuan

**Affiliations:** a Department of Laboratory and Equipment Management, Yanbian University, Yanbian 133002, People’s Republic of China

## Abstract

In the title compound, [Mn(C_9_H_6_N_3_O_2_)_2_(H_2_O)_4_]·10H_2_O, the Mn^II^ ion is coordinated by two N atoms from two 4-(4*H*-1,2,4-triazol-4-yl)benzoate ligands and four water mol­ecules in a distorted octa­hedral geometry. The Mn^II^ ion and two coordinated water mol­ecules lie on a twofold rotation axis. The water mol­ecules are involved in O—H⋯N and O—H⋯O hydrogen bonds with the triazole N atoms and carboxyl­ate O atoms, yielding a three-dimensional supra­molecular network. π–π inter­actions between the benzene rings [centroid–centroid distance = 3.836 (9) Å] are observed.

## Related literature

For general background to the applications of coordination polymers, see: Guo *et al.* (2009 ▶); Wang *et al.* (2009 ▶); Zang *et al.* (2006 ▶). For a related structure, see: Wang (2011 ▶).
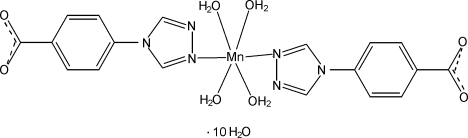


## Experimental

### 

#### Crystal data

[Mn(C_9_H_6_N_3_O_2_)_2_(H_2_O)_4_]·10H_2_O
*M* *_r_* = 683.50
Monoclinic, 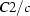

*a* = 25.9966 (13) Å
*b* = 7.9393 (4) Å
*c* = 16.8495 (9) Å
β = 112.214 (1)°
*V* = 3219.5 (3) Å^3^
*Z* = 4
Mo *K*α radiation
μ = 0.49 mm^−1^
*T* = 76 K
0.28 × 0.23 × 0.20 mm

#### Data collection

Bruker APEX CCD diffractometer
Absorption correction: multi-scan (*SADABS*; Bruker, 2001 ▶) *T*_min_ = 0.85, *T*_max_ = 0.91
8592 measured reflections
3189 independent reflections
2760 reflections with *I* > 2σ(*I*)
*R*_int_ = 0.023

#### Refinement

*R*[*F* ^2^ > 2σ(*F* ^2^)] = 0.029
*wR*(*F* ^2^) = 0.073
*S* = 0.99
3189 reflections
238 parameters
14 restraints
H atoms treated by a mixture of independent and constrained refinement
Δρ_max_ = 0.27 e Å^−3^
Δρ_min_ = −0.22 e Å^−3^

### 

Data collection: *SMART* (Bruker, 2007 ▶); cell refinement: *SAINT* (Bruker, 2007 ▶); data reduction: *SAINT*; program(s) used to solve structure: *SHELXS97* (Sheldrick, 2008 ▶); program(s) used to refine structure: *SHELXL97* (Sheldrick, 2008 ▶); molecular graphics: *SHELXTL* (Sheldrick, 2008 ▶); software used to prepare material for publication: *SHELXTL*.

## Supplementary Material

Crystal structure: contains datablock(s) global, I. DOI: 10.1107/S1600536811025335/hy2445sup1.cif

Structure factors: contains datablock(s) I. DOI: 10.1107/S1600536811025335/hy2445Isup2.hkl

Additional supplementary materials:  crystallographic information; 3D view; checkCIF report

## Figures and Tables

**Table 1:** Hydrogen-bond geometry (Å, °)

*D*—H⋯*A*	*D*—H	H⋯*A*	*D*⋯*A*	*D*—H⋯*A*
O1*W*—H1*A*⋯O4*W*	0.82 (2)	1.94 (2)	2.7602 (17)	171 (2)
O1*W*—H1*B*⋯O5*W*	0.85 (2)	1.83 (2)	2.6724 (16)	169 (2)
O2*W*—H2*A*⋯O1^i^	0.84 (1)	1.87 (1)	2.6936 (15)	164 (2)
O3*W*—H3*A*⋯O1^ii^	0.85 (2)	1.91 (2)	2.7445 (15)	166 (2)
O4*W*—H4*A*⋯O2^iii^	0.85 (2)	1.95 (2)	2.7985 (15)	176 (2)
O4*W*—H4*B*⋯N2^iv^	0.82 (2)	2.17 (2)	2.9369 (17)	154 (2)
O5*W*—H5*A*⋯O2^v^	0.85 (2)	1.83 (2)	2.6765 (16)	171 (2)
O5*W*—H5*B*⋯O8*W*^ii^	0.83 (2)	1.90 (2)	2.7299 (18)	172 (2)
O6*W*—H6*A*⋯O7*W*^vi^	0.86 (2)	1.89 (2)	2.754 (2)	177 (2)
O6*W*—H6*B*⋯O5*W*^ii^	0.83 (2)	1.95 (2)	2.7828 (18)	173 (2)
O7*W*—H7*A*⋯O6*W*	0.84 (2)	1.89 (2)	2.7256 (19)	171 (2)
O7*W*—H7*B*⋯O8*W*^vi^	0.83 (2)	1.94 (2)	2.7605 (18)	171 (2)
O8*W*—H8*A*⋯O1	0.84 (2)	1.92 (2)	2.7564 (16)	173 (2)
O8*W*—H8*B*⋯O4*W*^i^	0.86 (2)	1.91 (2)	2.7616 (17)	172 (2)

Symmetry codes: (i) 
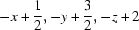
; (ii) 
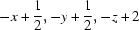
; (iii) 
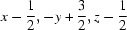
; (iv) 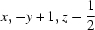; (v) 
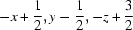
; (vi) 
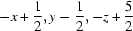
.

**Fig. 1.**
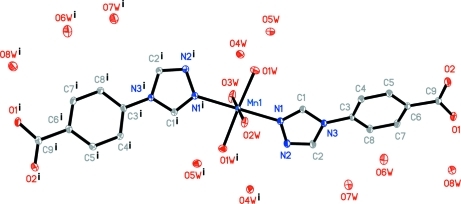
The molecular structure of the title compound. Displacement ellipsoids are drawn at the 30% probability level. [Symmetry code: (i) -x, y, 3/2-z.]

**Fig. 2.**
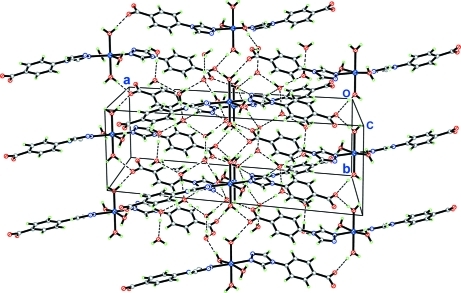
View of the three-dimensional network of the title compound, built by hydrogen bonds (dashed lines).

## supplementary crystallographic information

### Comment

The construction of novel coordination polymers is the current interest in the field of supramolecular chemistry and crystal engineering, not only for their interesting topologies and crystal packing motifs but also for their potential applications as functional materials (Wang *et al.*, 2009; Zang *et al.*, 2006). As an important family of multidentate O-donor ligands, organic aromatic carboxylate ligands have been extensively employed in the preparation of metal-organic complexes (Guo *et al.*, 2009). In this paper, we selected 4-(1,2,4-triazol-4-yl)benzoic acid as an organic carboxylate ligand, generating the title compound, which is reported here.

In the title compound, the Mn^II^ ions lies on a twofold rotation axis and is approximately octahedrally coordinated by two N atoms from two 4-(1,2,4-triazol-4-yl)benzoate ligands and four water molecules, two of which lie on the twofold rotation axis (Fig. 1). The Mn—N and Mn—O bond lengths and the O—Mn—O and N—Mn—O bond angles are comparable to those found in the other crystallographically characterized Mn(II) complexes (Wang, 2011). The water molecules are involved in O—H···N and O—H···O hydrogen bonds with the triazole N atoms and carboxylate O atoms (Table 1), yielding a three-dimensional supramolecular network (Fig. 2). π–π interactions between the benzene rings [centroid–centroid distance = 3.836 (9) Å] are observed.

### Experimental

The synthesis was performed under hydrothermal conditions. A mixture of Mn(CH_3_COO)_2_.4H_2_O (0.2 mmol, 0.049 g), 4-(1,2,4-triazol-4-yl)benzoic acid (0.4 mmol, 0.075 g), NaOH (0.4 mmol, 0.016 g) and H_2_O (15 ml) in a 25 ml stainless steel reactor with a Teflon liner was heated from 293 to 443 K in 2 h and a constant temperature was maintained at 443 K for 72 h. After the mixture was cooled to 298 K, purple crystals of the title compound were obtained from the reaction.

### Refinement

H atoms on C atoms were positioned geometrically and refined as riding atoms, with C—H = 0.95 Å and *U*_iso_(H) = 1.2*U*_eq_(C). H atoms of water molecules were located in a difference Fourier map and refined with an O—H distance restraint of 0.85 (2) Å and with *U*_iso_(H) = 1.5*U*_eq_(O).

### Crystal data

**Table:**

[Mn(C_9_H_6_N_3_O_2_)_2_(H_2_O)_4_]·10H_2_O	*F*(000) = 1436
*M**_r_* = 683.50	*D*_x_ = 1.410 Mg m^−^^3^
Monoclinic, *C*2/*c*	Mo *K*α radiation, λ = 0.71073 Å
Hall symbol: -C 2yc	Cell parameters from 3198 reflections
*a* = 25.9966 (13) Å	θ = 1.0–26.1°
*b* = 7.9393 (4) Å	µ = 0.49 mm^−^^1^
*c* = 16.8495 (9) Å	*T* = 76 K
β = 112.214 (1)°	Block, purple
*V* = 3219.5 (3) Å^3^	0.28 × 0.23 × 0.20 mm
*Z* = 4	

### Data collection

**Table:**

Bruker APEX CCD diffractometer	3189 independent reflections
Radiation source: fine-focus sealed tube	2760 reflections with *I* > 2σ(*I*)
graphite	*R*_int_ = 0.023
φ and ω scans	θ_max_ = 26.1°, θ_min_ = 1.7°
Absorption correction: multi-scan (*SADABS*; Bruker, 2001)	*h* = −19→32
*T*_min_ = 0.85, *T*_max_ = 0.91	*k* = −8→9
8592 measured reflections	*l* = −20→20

### Refinement

**Table:**

Refinement on *F*^2^	Primary atom site location: structure-invariant direct methods
Least-squares matrix: full	Secondary atom site location: difference Fourier map
*R*[*F*^2^ > 2σ(*F*^2^)] = 0.029	Hydrogen site location: inferred from neighbouring sites
*wR*(*F*^2^) = 0.073	H atoms treated by a mixture of independent and constrained refinement
*S* = 0.99	*w* = 1/[σ^2^(*F*_o_^2^) + (0.036*P*)^2^ + 1.9266*P*] where *P* = (*F*_o_^2^ + 2*F*_c_^2^)/3
3189 reflections	(Δ/σ)_max_ = 0.008
238 parameters	Δρ_max_ = 0.27 e Å^−^^3^
14 restraints	Δρ_min_ = −0.22 e Å^−^^3^

### Fractional atomic coordinates and isotropic or equivalent isotropic displacement parameters (Å^2^)

**Table:**

	*x*	*y*	*z*	*U*_iso_*/*U*_eq_	
C1	0.12836 (6)	0.4382 (2)	0.87216 (9)	0.0226 (3)	
H1	0.1368	0.4674	0.8237	0.027*	
C2	0.13557 (6)	0.3880 (2)	1.00119 (10)	0.0263 (4)	
H2	0.1504	0.3752	1.0617	0.032*	
C3	0.22362 (6)	0.48018 (19)	0.98458 (9)	0.0195 (3)	
C4	0.24336 (6)	0.5736 (2)	0.93267 (9)	0.0229 (3)	
H4	0.2190	0.6071	0.8770	0.027*	
C5	0.29901 (6)	0.6175 (2)	0.96291 (10)	0.0232 (3)	
H5	0.3129	0.6801	0.9272	0.028*	
C6	0.33496 (6)	0.57157 (18)	1.04481 (9)	0.0200 (3)	
C7	0.31413 (6)	0.47742 (19)	1.09554 (9)	0.0229 (3)	
H7	0.3383	0.4451	1.1515	0.027*	
C8	0.25869 (6)	0.42984 (19)	1.06575 (10)	0.0231 (3)	
H8	0.2450	0.3638	1.1005	0.028*	
C9	0.39483 (6)	0.62809 (19)	1.07889 (10)	0.0210 (3)	
N1	0.07926 (5)	0.39357 (16)	0.86873 (8)	0.0218 (3)	
N2	0.08381 (5)	0.36106 (18)	0.95205 (8)	0.0266 (3)	
N3	0.16569 (5)	0.43720 (16)	0.95412 (8)	0.0203 (3)	
O1	0.42246 (4)	0.60776 (13)	1.15861 (7)	0.0245 (2)	
O2	0.41384 (4)	0.69525 (16)	1.02887 (7)	0.0324 (3)	
Mn1	0.0000	0.39602 (4)	0.7500	0.01689 (10)	
O1W	0.05118 (5)	0.41965 (15)	0.67562 (7)	0.0284 (3)	
H1A	0.0417 (8)	0.493 (2)	0.6382 (11)	0.043*	
H1B	0.0664 (8)	0.341 (2)	0.6578 (12)	0.043*	
O2W	0.0000	0.6681 (2)	0.7500	0.0256 (3)	
H2A	0.0229 (7)	0.732 (2)	0.7865 (11)	0.038*	
O3W	0.0000	0.1260 (2)	0.7500	0.0381 (4)	
H3A	0.0248 (8)	0.065 (3)	0.7856 (12)	0.057*	
O4W	0.02208 (5)	0.69006 (15)	0.56446 (7)	0.0278 (3)	
H4A	−0.0110 (6)	0.724 (2)	0.5514 (12)	0.042*	
H4B	0.0298 (8)	0.688 (3)	0.5212 (11)	0.042*	
O5W	0.10937 (5)	0.17426 (16)	0.63970 (8)	0.0301 (3)	
H5A	0.1048 (8)	0.174 (3)	0.5870 (10)	0.045*	
H5B	0.1054 (9)	0.075 (2)	0.6528 (13)	0.045*	
O6W	0.29468 (5)	0.16683 (18)	1.25059 (9)	0.0427 (3)	
H6A	0.2976 (10)	0.062 (2)	1.2643 (15)	0.064*	
H6B	0.3218 (8)	0.217 (3)	1.2862 (13)	0.064*	
O7W	0.19555 (5)	0.32849 (17)	1.21048 (8)	0.0363 (3)	
H7A	0.2252 (7)	0.272 (3)	1.2263 (14)	0.054*	
H7B	0.1697 (8)	0.268 (3)	1.2113 (14)	0.054*	
O8W	0.39715 (5)	0.64318 (16)	1.30252 (8)	0.0318 (3)	
H8A	0.4037 (8)	0.640 (3)	1.2571 (11)	0.048*	
H8B	0.4240 (7)	0.696 (3)	1.3407 (12)	0.048*	

### Atomic displacement parameters (Å^2^)

**Table:**

	*U*^11^	*U*^22^	*U*^33^	*U*^12^	*U*^13^	*U*^23^
C1	0.0166 (7)	0.0320 (8)	0.0177 (7)	−0.0016 (6)	0.0046 (6)	0.0000 (6)
C2	0.0190 (8)	0.0397 (9)	0.0194 (8)	−0.0045 (7)	0.0061 (6)	0.0039 (7)
C3	0.0131 (7)	0.0232 (8)	0.0205 (7)	−0.0022 (6)	0.0043 (6)	−0.0030 (6)
C4	0.0179 (7)	0.0326 (9)	0.0153 (7)	−0.0009 (6)	0.0030 (6)	0.0011 (6)
C5	0.0192 (8)	0.0307 (9)	0.0202 (8)	−0.0038 (6)	0.0081 (6)	0.0007 (6)
C6	0.0163 (7)	0.0214 (8)	0.0213 (7)	−0.0010 (6)	0.0059 (6)	−0.0036 (6)
C7	0.0181 (7)	0.0271 (8)	0.0187 (7)	−0.0005 (6)	0.0015 (6)	0.0020 (6)
C8	0.0197 (8)	0.0272 (8)	0.0208 (8)	−0.0035 (6)	0.0059 (6)	0.0049 (6)
C9	0.0171 (7)	0.0219 (8)	0.0232 (8)	−0.0008 (6)	0.0065 (6)	−0.0032 (6)
N1	0.0169 (6)	0.0286 (7)	0.0188 (6)	−0.0017 (5)	0.0055 (5)	0.0004 (5)
N2	0.0180 (6)	0.0403 (8)	0.0199 (7)	−0.0037 (6)	0.0054 (5)	0.0032 (6)
N3	0.0145 (6)	0.0272 (7)	0.0174 (6)	−0.0024 (5)	0.0040 (5)	0.0001 (5)
O1	0.0164 (5)	0.0294 (6)	0.0218 (6)	−0.0021 (4)	0.0005 (4)	−0.0002 (5)
O2	0.0201 (6)	0.0497 (8)	0.0257 (6)	−0.0113 (5)	0.0070 (5)	−0.0001 (5)
Mn1	0.01246 (16)	0.01903 (17)	0.01757 (17)	0.000	0.00386 (12)	0.000
O1W	0.0300 (6)	0.0310 (7)	0.0296 (6)	0.0080 (5)	0.0173 (5)	0.0054 (5)
O2W	0.0204 (8)	0.0201 (8)	0.0269 (9)	0.000	−0.0017 (7)	0.000
O3W	0.0310 (10)	0.0205 (9)	0.0420 (11)	0.000	−0.0100 (8)	0.000
O4W	0.0209 (6)	0.0408 (7)	0.0226 (6)	0.0057 (5)	0.0092 (5)	0.0024 (5)
O5W	0.0345 (7)	0.0308 (6)	0.0286 (6)	0.0019 (5)	0.0161 (5)	−0.0011 (5)
O6W	0.0335 (7)	0.0381 (8)	0.0489 (9)	−0.0018 (6)	0.0070 (6)	−0.0009 (7)
O7W	0.0291 (7)	0.0362 (7)	0.0402 (7)	−0.0020 (6)	0.0092 (6)	0.0045 (6)
O8W	0.0277 (7)	0.0374 (7)	0.0319 (7)	−0.0053 (5)	0.0131 (5)	−0.0047 (6)

### Geometric parameters (Å, °)

**Table:**

C1—N1	1.3049 (19)	N1—N2	1.3877 (17)
C1—N3	1.3549 (19)	Mn1—N1	2.2652 (12)
C1—H1	0.9500	Mn1—O3W	2.1438 (17)
C2—N2	1.304 (2)	Mn1—O1W	2.1534 (11)
C2—N3	1.365 (2)	Mn1—O2W	2.1598 (16)
C2—H2	0.9500	O1W—H1A	0.82 (2)
C3—C4	1.385 (2)	O1W—H1B	0.85 (2)
C3—C8	1.385 (2)	O2W—H2A	0.84 (1)
C3—N3	1.4363 (18)	O3W—H3A	0.85 (2)
C4—C5	1.384 (2)	O4W—H4A	0.85 (2)
C4—H4	0.9500	O4W—H4B	0.82 (2)
C5—C6	1.391 (2)	O5W—H5A	0.85 (2)
C5—H5	0.9500	O5W—H5B	0.83 (2)
C6—C7	1.390 (2)	O6W—H6A	0.86 (2)
C6—C9	1.509 (2)	O6W—H6B	0.83 (2)
C7—C8	1.387 (2)	O7W—H7A	0.84 (2)
C7—H7	0.9500	O7W—H7B	0.83 (2)
C8—H8	0.9500	O8W—H8A	0.84 (2)
C9—O2	1.2466 (18)	O8W—H8B	0.86 (2)
C9—O1	1.2715 (18)		
			
N1—C1—N3	110.81 (13)	C2—N2—N1	106.54 (12)
N1—C1—H1	124.6	C1—N3—C2	104.28 (12)
N3—C1—H1	124.6	C1—N3—C3	127.81 (12)
N2—C2—N3	111.04 (14)	C2—N3—C3	127.91 (13)
N2—C2—H2	124.5	O3W—Mn1—O1W	95.00 (3)
N3—C2—H2	124.5	O3W—Mn1—O1W^i^	95.00 (3)
C4—C3—C8	121.02 (13)	O1W—Mn1—O1W^i^	170.01 (7)
C4—C3—N3	119.36 (13)	O3W—Mn1—O2W	180.000 (1)
C8—C3—N3	119.61 (13)	O1W—Mn1—O2W	85.00 (3)
C5—C4—C3	119.19 (14)	O1W^i^—Mn1—O2W	85.00 (3)
C5—C4—H4	120.4	O3W—Mn1—N1	89.51 (3)
C3—C4—H4	120.4	O1W—Mn1—N1	87.64 (4)
C4—C5—C6	121.04 (14)	O1W^i^—Mn1—N1	92.44 (4)
C4—C5—H5	119.5	O2W—Mn1—N1	90.49 (3)
C6—C5—H5	119.5	O3W—Mn1—N1^i^	89.51 (3)
C7—C6—C5	118.62 (13)	O1W—Mn1—N1^i^	92.44 (4)
C7—C6—C9	120.75 (13)	O1W^i^—Mn1—N1^i^	87.64 (4)
C5—C6—C9	120.59 (13)	O2W—Mn1—N1^i^	90.49 (3)
C8—C7—C6	121.12 (14)	N1—Mn1—N1^i^	179.02 (7)
C8—C7—H7	119.4	Mn1—O1W—H1A	115.8 (14)
C6—C7—H7	119.4	Mn1—O1W—H1B	127.7 (14)
C3—C8—C7	118.98 (14)	H1A—O1W—H1B	107.0 (19)
C3—C8—H8	120.5	Mn1—O2W—H2A	127.0 (13)
C7—C8—H8	120.5	Mn1—O3W—H3A	125.0 (15)
O2—C9—O1	123.96 (14)	H4A—O4W—H4B	109.8 (19)
O2—C9—C6	119.03 (13)	H5A—O5W—H5B	107 (2)
O1—C9—C6	116.98 (13)	H6A—O6W—H6B	108 (2)
C1—N1—N2	107.33 (12)	H7A—O7W—H7B	110 (2)
C1—N1—Mn1	125.78 (10)	H8A—O8W—H8B	108 (2)
N2—N1—Mn1	126.61 (9)		
			
C8—C3—C4—C5	−0.3 (2)	Mn1—N1—N2—C2	−174.12 (11)
N3—C3—C4—C5	178.76 (14)	N1—C1—N3—C2	0.07 (18)
C3—C4—C5—C6	−1.0 (2)	N1—C1—N3—C3	−179.24 (14)
C4—C5—C6—C7	1.2 (2)	N2—C2—N3—C1	−0.05 (18)
C4—C5—C6—C9	−176.69 (14)	N2—C2—N3—C3	179.26 (14)
C5—C6—C7—C8	−0.1 (2)	C4—C3—N3—C1	18.3 (2)
C9—C6—C7—C8	177.78 (14)	C8—C3—N3—C1	−162.65 (15)
C4—C3—C8—C7	1.3 (2)	C4—C3—N3—C2	−160.86 (16)
N3—C3—C8—C7	−177.70 (14)	C8—C3—N3—C2	18.2 (2)
C6—C7—C8—C3	−1.2 (2)	C1—N1—Mn1—O3W	109.92 (13)
C7—C6—C9—O2	171.81 (15)	N2—N1—Mn1—O3W	−76.98 (12)
C5—C6—C9—O2	−10.4 (2)	C1—N1—Mn1—O1W	14.90 (13)
C7—C6—C9—O1	−10.1 (2)	N2—N1—Mn1—O1W	−172.00 (12)
C5—C6—C9—O1	167.71 (14)	C1—N1—Mn1—O1W^i^	−155.10 (13)
N3—C1—N1—N2	−0.06 (18)	N2—N1—Mn1—O1W^i^	18.00 (12)
N3—C1—N1—Mn1	174.15 (10)	C1—N1—Mn1—O2W	−70.08 (13)
N3—C2—N2—N1	0.02 (19)	N2—N1—Mn1—O2W	103.02 (12)
C1—N1—N2—C2	0.02 (17)		

Symmetry codes: (i) −*x*, *y*, −*z*+3/2.

### Hydrogen-bond geometry (Å, °)

**Table:**

*D*—H···*A*	*D*—H	H···*A*	*D*···*A*	*D*—H···*A*
O1W—H1A···O4W	0.82 (2)	1.94 (2)	2.7602 (17)	171 (2)
O1W—H1B···O5W	0.85 (2)	1.83 (2)	2.6724 (16)	169 (2)
O2W—H2A···O1^ii^	0.84 (1)	1.87 (1)	2.6936 (15)	164 (2)
O3W—H3A···O1^iii^	0.85 (2)	1.91 (2)	2.7445 (15)	166 (2)
O4W—H4A···O2^iv^	0.85 (2)	1.95 (2)	2.7985 (15)	176 (2)
O4W—H4B···N2^v^	0.82 (2)	2.17 (2)	2.9369 (17)	154 (2)
O5W—H5A···O2^vi^	0.85 (2)	1.83 (2)	2.6765 (16)	171 (2)
O5W—H5B···O8W^iii^	0.83 (2)	1.90 (2)	2.7299 (18)	172 (2)
O6W—H6A···O7W^vii^	0.86 (2)	1.89 (2)	2.754 (2)	177 (2)
O6W—H6B···O5W^iii^	0.83 (2)	1.95 (2)	2.7828 (18)	173 (2)
O7W—H7A···O6W	0.84 (2)	1.89 (2)	2.7256 (19)	171 (2)
O7W—H7B···O8W^vii^	0.83 (2)	1.94 (2)	2.7605 (18)	171 (2)
O8W—H8A···O1	0.84 (2)	1.92 (2)	2.7564 (16)	173 (2)
O8W—H8B···O4W^ii^	0.86 (2)	1.91 (2)	2.7616 (17)	172 (2)

Symmetry codes: (ii) −*x*+1/2, −*y*+3/2, −*z*+2; (iii) −*x*+1/2, −*y*+1/2, −*z*+2; (iv) *x*−1/2, −*y*+3/2, *z*−1/2; (v) *x*, −*y*+1, *z*−1/2; (vi) −*x*+1/2, *y*−1/2, −*z*+3/2; (vii) −*x*+1/2, *y*−1/2, −*z*+5/2.
